# Jieyu Anshen Granule, a Chinese Herbal Formulation, Exerts Effects on Poststroke Depression in Rats

**DOI:** 10.1155/2020/7469068

**Published:** 2020-02-23

**Authors:** Yuan Du, Jian Ruan, Leiming Zhang, Fenghua Fu

**Affiliations:** ^1^Key Laboratory of Molecular Pharmacology and Drug Evaluation (Ministry of Education of China), School of Pharmacy, Yantai University, Yantai 264005, China; ^2^Yantai Center for Food and Drug Control, Yantai 264000, China

## Abstract

Jieyu Anshen granule (JY) is a traditional Chinese medicine formula for treating depression and anxiety. The aim of the study was to observe the effects of JY on poststroke depression (PSD) and investigate the underlying mechanism. PSD rat model was developed by middle cerebral artery occlusion following chronic unpredictable mild stress in conjunction with isolation rearing. We performed behavioral tests, Western blot, ELISA, and BrdU/NeuN staining. Treatment with JY showed significant antidepressant effect in open-field and sucrose preference tests, as well as significant improvement in beam-walking, cylinder, grip strength, and water maze tests. In addition, treatment with JY could restore the levels of neurotransmitters and decrease the levels of hormone and inflammation cytokines in serum and brain. Treatment with JY also showed significant regulation in the expression of neurotransmitter receptors and NF-*κ*B/I*κ*B-*α* signaling in the prefrontal cortex and hippocampus. Moreover, the numbers of newborn neurons in the hippocampus were increased by treatment with JY. Our results suggest that JY could ameliorate PSD and improve the neurological and cognitive functions. The antidepressive effect may be associated with the modulation of JY on monoamine system, neuroendocrine, neuroinflammation, and neurogenesis.

## 1. Introduction

Poststroke depression (PSD), which is different from general depression, is an extremely frequently neuropsychiatric disorder following ischaemic stroke, and common mood symptoms after stroke include anxiety and feelings of despair as well as anhedonia [1]. One-third of stroke patients suffer from depression, and depression negatively affects patients' ability to engage in rehabilitation therapies [2].

The pathophysiology of PSD is associated with a complex network of interrelated regulatory factors including the hypothalamic-pituitary-adrenal (HPA) axis, monoamine neurotransmitters, neuroinflammation, and neurogenesis [3]. Selective serotonin reuptake inhibitors (SSRIs) are the first choice for the pharmacological prevention and therapy of PSD [2, 4]. Patients taking antidepressant drugs often experience high relapse rates and a variety of side effects, such as nausea, headaches, somnolence, and dry mouth [5, 6].

Traditional Chinese medicine (TCM) is one of the commonly used complementary and alternative medicine therapies for depression, while a formula contains several herbs (Chaihu, Gancao, Fuling, Suanzaoren, Yujin, Baizhu, Yuanzhi, Shichangpu, Banxia, etc.) with a specific proportion interacting with each other to improve therapeutic effects and to reduce toxicities [7, 8]. TCM or its major constituents are often used in clinical practice in China for treating depression; for example, polyphenols may show antidepressant effects through normalizing HPA hyperactivity [9]. Jieyu Anshen granules (JY) are a classical TCM with antidepressant activity that have been recognized by the China Food and Drug Administration [10]. The use of JY alone or in combination with additional antidepressants has already been widely implemented in China as a means of treating anxiety and depression [11]. However, it is not clear whether JY can be effective for PSD. Thus, a rat model of PSD was established using chronic unpredictable mild stress (CUMS) following middle cerebral artery occlusion (MCAO); then effects of JY were observed and underlying mechanism was investigated.

## 2. Materials and Methods

### 2.1. Reagents and Drugs

JY (batch number: 20150501) was purchased from Baixing Tang Pharmaceutical Co. Ltd (Jilin, China), and it was composed of a total of 16 herbs (Table 1). Citalopram (batch number: 170902050) was purchased from Lundbeck Pharmaceutical Co. Ltd (Copenhagen, Denmark). 5-hydroxytryptamine (5-HT) and dopamine (DA) standards were purchased from Sigma Co. Ltd, while norepinephrine (NE) standards were purchased from the Pharmaceutical and Biological Products Institute of China. Primary antibodies against 5-hydroxytryptamine receptor 1A (5-HT_1A_R), alpha-2 adrenergic receptor (ADR*α*2), nuclear factor kappa B (NF-*κ*B), inhibitor of nuclear factor kappa B *α* (I*κ*B-*α*), glucocorticoid receptor (GR), *β*-actin, and secondary antibody were purchased from Santa Cruz Biotechnology Co. Ltd (Dallas, TX, USA) and Beyotime Biotechnology Co. Ltd (Shanghai, China) for Western blot, respectively. Primary and secondary antibodies against 5-Bromo-2-deoxyuridine (BrdU) were purchased from Bioss (Beijing, China) and Cwbio (Beijing, China) Co. Ltd. Primary and secondary antibodies against neuronal nuclei (NeuN) were purchased from Abcam (Cambridge, USA) and Cwbio (Beijing, China) Co. Ltd. ELISA and Bradford assay kits were purchased from Xinbo Sheng (Shenzhen, China) and Beyotime Biotechnology (Shanghai, China), respectively. All chemicals were of analytical grade.

### 2.2. Animals

Male Sprague-Dawley rats (240–260 g, 7–9 weeks) provided by Pengyue Animal Co. Ltd (License No. SCXK 20140007) had ad libitum food/water access while housed at 22 ± 2°C with 55 ± 5% humidity, as well as a 12 hour light/dark cycle. All animals had 1 week to acclimatize prior to study initiation. All studies were consistent with the guide from the National Institute of Health and received approval from the Ethical Committee of Yantai University.

### 2.3. Experimental Design and Drug Administration

75 rats were anaesthetized with sodium pentobarbital (35 mg/kg, i.p.), the left common carotid artery was exposed, and a small incision was made near the bifurcation of the external carotid artery and internal carotid artery (ICA). The 4/0 nylon monofilament suture (Xinong Biotech Co. Ltd., Beijing, China), which was prepared, heat-blunted at the tip to a diameter of 0.36 mm, and coated with poly-L-lysine, was then gently inserted into the artery from the ICA incision, with the length of 18.0 ± 0.5 mm. The internal temperature of rats was maintained at 37°C during the operation. Rats surviving with neurological scores ≥1 and <4 were randomized into 5 groups: CON (Sham operated controls; *n* = 10), MCAO + CUMS (MCAO, then CUMS for 18 days; *n* = 10), CIT10 (MCAO + CUMS + Citalopram 10 mg/kg; *n* = 10), JY1 (MCAO + CUMS + JY 1 g/kg; *n* = 10), and JY3 (MCAO + CUMS + JY 3 g/kg; *n* = 10).

CUMS consisted of a total of 7 unique stressors: swimming in 4°C water (5 min), 45° cage tilt (17 h), water deprivation (18 h, after which empty water bottles were given for 1 h), deprivation of food and water (20 h, followed by a sucrose preference test), soiled cages (200 ml water mixed into 100 g sawdust bedding; 21 h), paired caging (2 h), and overnight illumination (no darkness for 36 h). Neurological scores were determined 24 h after MCAO, then stressors were administered to rats in a random order for the following 18 days [12]. The rats were isolation-reared (one per cage) except CON group.

**Figure 1:**
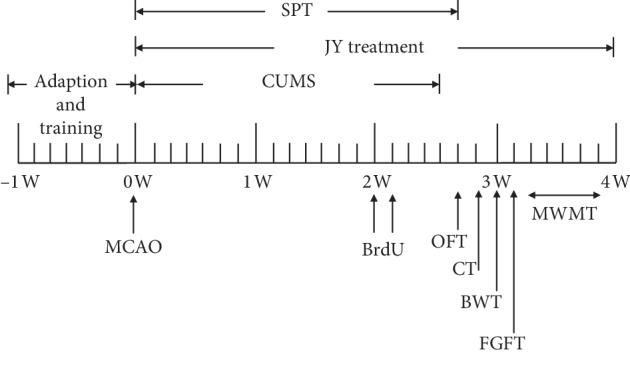
Study schedule.

From the second day after MCAO, rats in the JY groups were administered JY (1 or 3 g/kg) daily for 4 weeks, while rats in the CIT10 group were administered citalopram (10 mg/kg) on the same schedule. Both JY and citalopram were administered intragastrically a volume of 1 mL/kg. Study design is illustrated in Figure 1.

### 2.4. Procedure for Evaluation of Depressive-Like Behavior in Rats

#### 2.4.1. Sucrose Preference Test

Reward behavior in rats was assessed via sucrose preference test (SPT). Animals were deprived of food/water for 20 h, after which 2 preweighed bottles were put into the cage for 1 h. One bottle was filled with water, while the other contained 1% sucrose. Between trials, bottle position was randomized, and within each trial bottle positions were identical for all rats. After the 1 h period, bottles were removed and weighed. This test was performed both at baseline and following treatment, and sucrose preference was calculated as follows: sucrose preference = sucrose intake (g)/sucrose intake (g) + water intake (g) [12].

#### 2.4.2. Open-Field Test

Despair of rats was evaluated by open-field test (OFT). For this examination, rats were put into an open-topped cylindrical box with black walls (height, 40 cm; diameter, 75 cm). The bottom of the box was marked in 25 equally sized block sections. Rat locomotion and rearing activity were then monitored, with locomotor activity being quantified based on the number of blocks crossed and rearing activity being assessed based on how many times rats stood on their hind legs. Rats were each assessed one time for 3 min (isolation) [13].

### 2.5. Procedure for Evaluation of Neurological and Cognitive Function in Rats

#### 2.5.1. Beam-Walking Test

Rat coordination and motor movement integration was evaluated by beam-walking test (BWT) [14]. Before MCAO, rats underwent two training sessions per day for 2 days. During all sessions, a wooden beam (2.5 cm × 120 cm) was used, with a 30 × 30 × 30 wooden box placed on the other side to encourage rats to cross the beam. For training, rats were positioned on the center or starting points on the beam during the 1^st^ and 2^nd^ trials, respectively. Cushions were present under the beam to prevent any fall-induced injury to animals. Crossing time was determined as the time between when a forepaw was first extended onto the beam and when a forepaw was first extended into the wooden box, with a maximum cut-off time of 60 s.

#### 2.5.2. Cylinder Test

Asymmetry in forelimb use was evaluated by cylinder test (CT). For this assay, a transparent glass cylinder (30 cm high, 20 cm in diameter) was used. Rats were placed into the cylinder, and 20 movements were recorded for each animal, after which we calculated forelimb asymmetry as follows: 100 × (ipsilateral forelimb use +1/2 both)/total forelimb use [15].

#### 2.5.3. Forelimb Grip Force Test

Muscular strength was evaluated by forelimb grip force test (FGFT), using a grip strength meter [16]. To measure this strength, rats were held by the tail over a grid in a position where they were able to reach the strength meter using their forepaws. Maximal grip strength was that at which rats were no longer able to grip the meter when they were pulled backwards away from it.

#### 2.5.4. Memory Water Maze Test

Spatial memory was evaluated by memory water maze test (MWMT), which is a standard approach for evaluating cognitive function [17]. For this test, we filled a pool, 1.2 m in diameter, with water and placed the study animals into the pool for 120 s during which time they sought to locate a hidden platform which was positioned 1 cm beneath the water surface. Surrounding the walls of the pool were specific visual cues to guide rats, including a horizontal tube on the north wall, an “X” on the south wall, an entrance door on the east wall, and a vertical tube on the west wall. Upon finding the hidden platform, rats were given 15 s during which they could rest on it. If they did not locate this platform within 60 s, they were put onto it for 15 s. Over a period of 4 days, rats received 4 total attempts to locate this platform. On the 5^th^ day this platform was removed and rats were given a 60 s performance assessment. The time spent in the quadrant where the platform had been located was used as a means of evaluating the degree to which rats were able to remember and navigate to the platform location.

### 2.6. Western Blotting

Prefrontal cortex and hippocampal tissue samples were washed with PBS and then lysed via the use of lysis buffer containing phenylmethylsulfonyl fluoride. Lysates were thoroughly homogenized and then spun for 10 minutes (12,000 ×g at 4°C), after which a Bradford assay was utilized to measure protein concentrations in supernatants. These samples were then boiled in 5x loading buffer, electrophoretically separated using 12 % SDS-PAGE gels, and transferred to PVDF membranes that were blocked for 4 h with 5% nonfat dried milk. Membranes were then probed overnight using 1 : 1000 dilutions of appropriate primary antibodies against 5-HT_1A_R, ADR*α*2, NF-*κ*B, I*κ*B-*α*, GR, and *β*-actin at 4°C. Blots were then probed using appropriate secondary antibodies and were developed with ECL reagents and a gel imager (GE, LAS4000). Densitometric quantification was utilized as a means of assessing protein levels, and samples were normalized to levels of *β*-actin contained therein [18].

### 2.7. Biochemical Assay

#### 2.7.1. Determination of Neurotransmitters

The levels of NE, DA, and 5-HT in hippocampal and prefrontal cortex samples were measured by HPLC using an ultraviolet detector, with an approach adapted from previous work [19]. Briefly, we suspended brain tissue using ice-cold 0.6 mol/L perchloric acid supplemented with 0.5 mmol/L EDTA as well as 0.8 mmol/L L-cysteine. These tissue homogenates then underwent 4°C centrifugation at 16,000 rpm for 20 minutes, and supernatants were filtered via 0.25 filtration into the chromatographic system. A Quaternary pump HPLC system (SHIMADZU: LC-20A, Shimadzu, Kyoto, Japan), 25 cm long by 4.6 mm in diameter, and a Cosmosil C 18 Column (5 mm particles) (Inertsil ODS-2) were used for all HPLC. The mobile phase contained 90% (v/v) 0.1 mol/L of a sodium acetate solution (0.1 mmol/L EDTA-2Na, pH 5.1), 10% (v/v) methanol. A 20 *μ*L injection volume was used, with a 1 mL/min flow rate and a 275 nm wavelength. DA, 5-HT, and NE retention times were, respectively, 5.02, 10.08, and 3.37 minutes.

#### 2.7.2. Determination of Cytokines and Hormones

Levels of adrenocorticotropic hormone (ACTH), corticosterone (CORT), tumor necrosis factor *α* (TNF-*α*), and interleukin-1*β* (IL-1*β*) were determined using ELISA kits based on the instructions.

### 2.8. Immunofluorescence and Confocal Detection

Rats were injected with BrdU (50 mg/kg, i.p.) twice a day on days 14-15. On day 28, rats were anaesthetized and fixed by being perfused with 4% paraformaldehyde. The brains were removed, and the 16 *μ*m thick dentate gyrus sections of hippocampus were incubated overnight at 4°C with primary antibodies (mouse anti-BrdU, 1 : 100; rabbit anti-NeuN, 1 : 1000) followed by incubation for 2 hours in secondary antibodies (for BrdU, FITC-conjugated goat anti-mouse IgG, 1 : 1000; for NeuN, Cy3-conjugated goat anti-rabbit IgG, 1 : 1000). The confocal microscope (Zeiss LSM800) was used to acquire the images of BrdU+, NeuN+, and BrdU+/NeuN+ cells.

### 2.9. Statistical Analysis

SPSS v.16.0 was used for all analyses, and data are expressed as means ± SEM. Two-way ANOVAs were used to compare SPT results, with treatment and day as the two factors. Other behavioral tests, Western blotting, and biochemical assays were performed by using one-way ANOVA via Tukey's post hoc comparison when comparing more than two groups. *P* < 0.05 was considered significant.

## 3. Results

### 3.1. JY Improved Depressive Behaviors in PSD Rats

**Figure 2:**
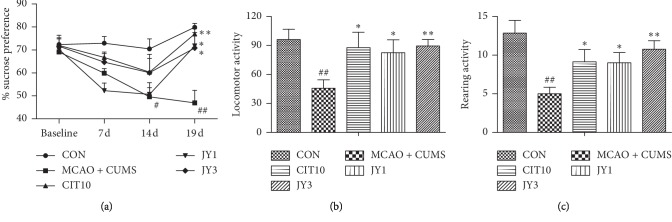
Effect of JY on depressive behaviors of PSD rats. (a) Sucrose preference test; (b) open-field test (locomotor activity); (c) open-field test (rearing activity). Data are means ± SEM; *n* = 7–10. ^#^*P* < 0.05;  ^##^*P* < 0.01 vs. CON. ^*∗*^*P* < 0.05;  ^*∗∗*^*P* < 0.01 vs. MCAO + CUMS.

The MCAO + CUMS rats significantly reduced the sucrose preference on day 14 compared to CON rats (*P* < 0.05), and the effect persisted until CUMS ended on day 19 (*P* < 0.01). Treatment effect on sucrose preference was significant [*F*_(4,180)_ = 12.91, *P* < 0.01], as were the effect of days of treatment [*F*_(3,180)_ = 13.43, *P* < 0.01] and the interaction between days and treatment [*F*_(12,180)_ = 3.03, *P* < 0.01]. The CIT10, JY1, and JY3 groups exhibited a significant increase in sucrose preference on day 19 compared to MCAO + CUMS rats (*P* < 0.05, *P* < 0.01) (Figure 2(a)). The MCAO + CUMS rats showed decreased locomotor [*F*_(4,45)_ = 3.44, *P* < 0.01] and rearing activity [*F*_(4,45)_ = 4.67, *P* < 0.01] compared to CON rats. The activities of locomotor and rearing were increased compared to MCAO + CUMS rats in the CIT10 and JY (JY1 and JY3) treatment groups (*P* < 0.05, *P* < 0.01) (Figures 2(b) and 2(c)). The results suggested that JY could significantly exert antidepressant effect on PSD rat model.

### 3.2. JY Improved Neurological and Cognitive Functions in PSD Rats

**Figure 3:**
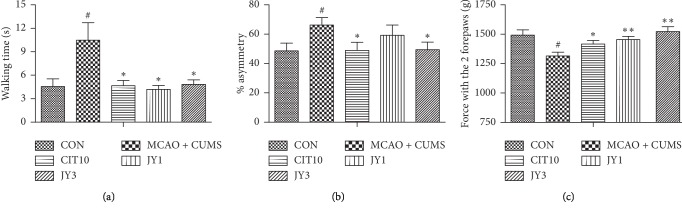
Effect of JY on neurological function of PSD rats. (a) Beam-walking test; (b) cylinder test; (c) forelimb grip force test. Data are means ± SEM; *n* = 7–10. ^#^*P* < 0.05 vs. CON. ^*∗*^*P* < 0.05,  ^*∗∗*^*P* < 0.01 vs. MCAO + CUMS.

**Figure 4:**
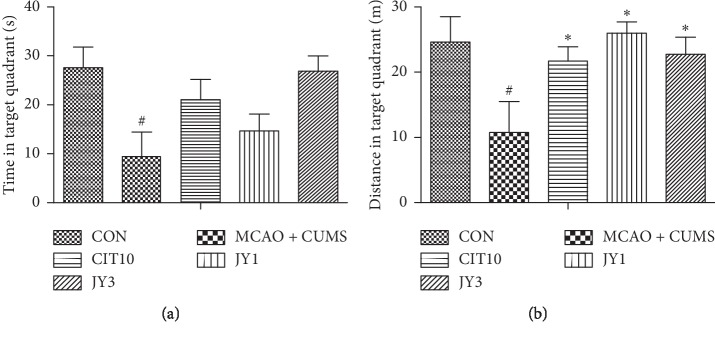
Effect of JY on time in target quadrant and distance in target quadrant of PSD rats. Data are means ± SEM; *n* = 7–10. ^#^*P* < 0.05 vs. CON. ^*∗*^*P* < 0.05 vs. MCAO + CUMS.

The MCAO + CUMS rats took longer time to cross the beam compared to CON rats [*F*_(4,40)_ = 5.04, *P* < 0.05]. The beam crossing time was significantly reduced in CIT10, JY1, and JY3 groups compared to MCAO + CUMS group (all *P* < 0.05) (Figure 3(a)). The MCAO + CUMS group showed increased asymmetry compared to CON group [*F*_(4,40)_ = 1.97, *P* < 0.05]. In contrast, the asymmetry was significantly reduced in both CIT10 and JY3 groups compared to MCAO + CUMS group (*P* < 0.05) (Figure 3(b)). The forepaw grip strength was decreased in MCAO + CUMS rats compared to CON rats [*F*_(4,40)_ = 5.08, *P* < 0.05], while CIT10, JY1, and JY3 groups significantly improved the strength compared to MCAO + CUMS group (*P* < 0.05, *P* < 0.01) (Figure 3(c)). The MCAO + CUMS rats significantly decreased the time [*F*_(4,35)_ = 2.71, *P* < 0.05] and distance [*F*_(4,35)_ = 3.50, *P* < 0.05] in the target quadrant compared to CON rats. Treatment of rats with JY or CIT10 significantly increased the distance and time in the target quadrant compared to MCAO + CUMS rats (all *P* < 0.05) (Figure 4). The results suggested that JY could significantly improve neurological and cognitive functions in PSD rats.

### 3.3. JY Increased the Levels of Neurotransmitters in Prefrontal Cortex, Hippocampus, Hypothalamus, and Striatum

As shown in Table 2, there was a significant increase in the levels of DA and 5-HT in prefrontal cortex of MCAO + CUMS rats compared to CON rats (all *P* < 0.01). Compared to MCAO + CUMS rats, the levels of DA and 5-HT were significantly increased in prefrontal cortex of CIT10 and JY1 groups (*P* < 0.05, *P* < 0.01). There was also significant differences in the levels of NE, DA, and 5-HT in hippocampus between MCAO + CUMS and CON rats (*P* < 0.05, *P* < 0.01). JY1 treatment significantly increased the levels of these neurotransmitters in the hippocampus compared to MCAO + CUMS rats (*P* < 0.05, *P* < 0.01), whereas CIT10 significantly increased the level of 5-HT (*P* < 0.05). Levels of these neurotransmitters in the hypothalamus of MCAO + CUMS rats were significantly decreased compared to CON rats (all *P* < 0.01). JY1 treatment significantly increased the levels of these neurotransmitters in the hypothalamus compared to the MCAO + CUMS rats (*P* < 0.05, *P* < 0.01), whereas CIT10 increased the levels of DA and 5-HT (*P* < 0.05, *P* < 0.01). The levels of these neurotransmitters in the striatum of MCAO + CUMS rats were significantly decreased compared to CON rats (all *P* < 0.05). Compared to MCAO + CUMS rats, the level of NE in the striatum of CIT10 and JY1 treated rats was significantly increased (*P* < 0.05, *P* < 0.01). The results suggested that the levels of neurotransmitters in brain could be increased by administration of JY on PSD rat model.

### 3.4. JY Regulated the Expression of 5-HT_1A_R and ADR*α*2 in Prefrontal Cortex and Hippocampus

**Figure 5:**
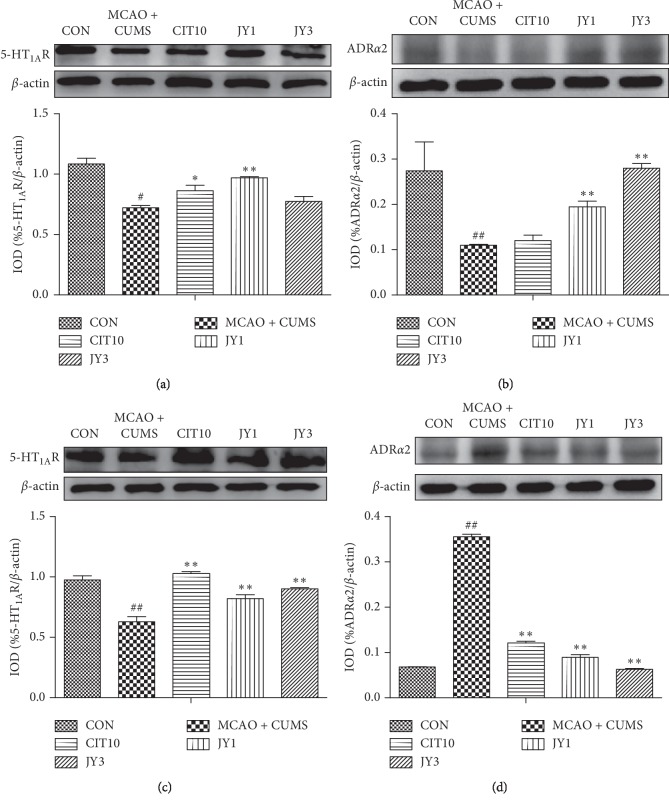
Effect of JY on the expression of 5-HT_1A_R and ADR*α*2 in prefrontal cortex and hippocampus of PSD rats. (a) 5-HT_1A_R expression in the prefrontal cortex; (b) ADR*α*2 expression in the prefrontal cortex. (c) 5-HT_1A_R expression in the hippocampus; (d) 5-HT_1A_R expression in the hippocampus. A representative blot and densitometric data for indicated bands are shown. Data are means ± SEM of triplicate experiments. ^#^*P* < 0.05,  ^##^*P* < 0.01 vs. CON. ^*∗*^*P* < 0.05,  ^*∗∗*^*P* < 0.01 vs. MCAO + CUMS.

The expression of 5-HT_1A_R was significantly reduced in prefrontal cortex in MCAO + CUMS rats compared to CON rats (*P* < 0.05). CIT10 and JY1 treatment significantly increased the expression of 5-HT_1A_R (*P* < 0.05, *P* < 0.01) (Figure 5(a)). The expression of ADR*α*2 was significantly reduced in prefrontal cortex in MCAO + CUMS rats compared to CON rats (*P* < 0.01). JY1 and JY3 treatment significantly increased the expression of 5-HT_1A_R (*P* < 0.01) (Figure 5(b)). The expression of hippocampal 5-HT_1A_R was significantly reduced in MCAO + CUMS rats compared to CON rats (*P* < 0.01). CIT10, JY1, and JY3 treatment significantly increased the expression of 5-HT_1A_R (*P* < 0.01) (Figure 5(c)). The expression of hippocampal ADR*α*2 was significantly increased in MCAO + CUMS rats compared to CON rats (*P* < 0.01), and CIT10, JY1, and JY3 significantly increased the expression of ADR*α*2 (*P* < 0.01) (Figure 5(d)). The results suggested that the levels of neurotransmitters in brain could be increased by administration of JY on PSD rat model.

### 3.5. JY Regulated the HPA Axis Activity and GR Expression in Hippocampus

**Figure 6:**
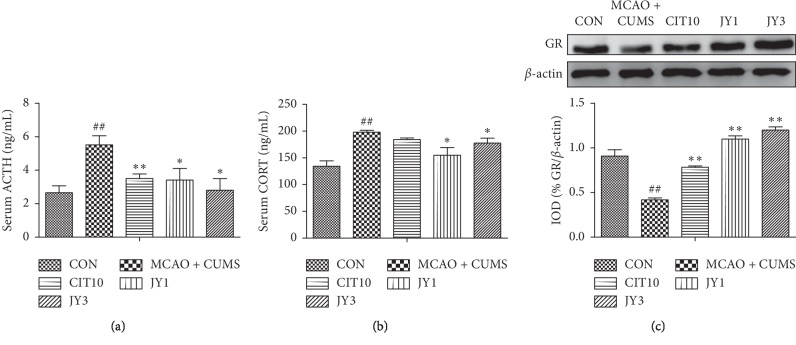
Effect of JY on the levels of serum ACTH and CORT, and the expression of GR in hippocampus of PSD rats. (a) Serum ACTH; (b) serum CORT; (c) GR expression in hippocampus. A representative blot and densitometric data for indicated bands are shown. Data are means ± SEM. *n* = 7–10. ^##^*P* < 0.01 vs. CON. ^*∗*^*P* < 0.05,  ^*∗∗*^*P* < 0.01 vs. MCAO + CUMS.

The level of serum ACTH was significantly increased in MCAO + CUMS rats compared to CON rats (*P* < 0.01). CIT10, JY1, and JY3 treatment significantly decreased the level of serum ACTH (*P* < 0.05, *P* < 0.01) (Figure 6(a)). The level of serum CORT was significantly increased in MCAO + CUMS rats compared to CON rats (*P* < 0.01). JY1 and JY3 treatment significantly decreased the level of serum CORT (*P* < 0.05, *P* < 0.01) (Figure 6(b)). The expression of hippocampal GR was significantly decreased in MCAO + CUMS rats compared to CON rats (*P* < 0.01). CIT10, JY1, and JY3 treatment significantly increased the expression of GR (*P* < 0.01) (Figure 6(c)). The results suggested that the dysfunction of HPA axis could be reversed by administration of JY on PSD rat model.

### 3.6. JY Decreased the Levels of TNF-*α* and IL-1*β* in Serum, Prefrontal Cortex, and Hippocampus

**Figure 7:**
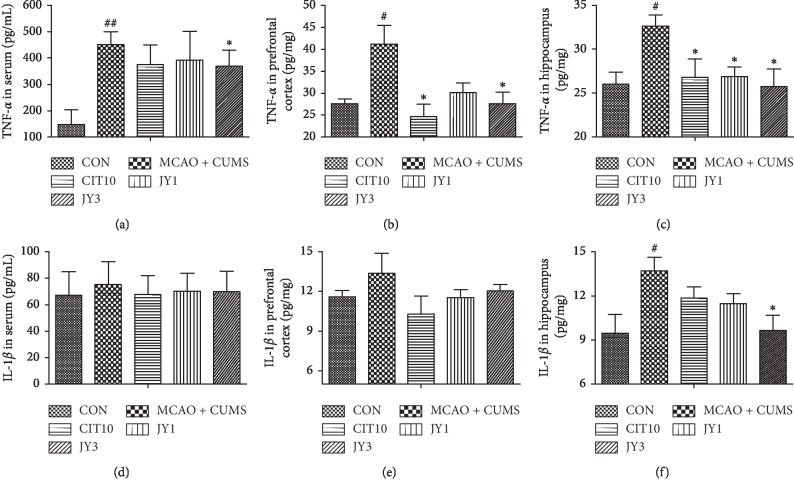
Effect of JY on the levels of TNF-*α* and IL-1*β* in serum, prefrontal cortex and hippocampus of PSD rats. (a) Serum TNF-*α*; (b) TNF-*α* in the prefrontal cortex; (c) TNF-*α* in hippocampus; (d) serum IL-1*β*; (e) IL-1*β* in the prefrontal cortex; (f) IL-1*β* in hippocampus. Data are means ± SEM; *n* = 7–10. ^#^*P* < 0.05,  ^##^*P* < 0.01 vs. CON. ^*∗*^*P* < 0.05 vs. MCAO + CUMS.

The level of TNF-*α* in serum, prefrontal cortex, and hippocampus was significantly increased in MCAO + CUMS rats compared to CON rats (*P* < 0.05, *P* < 0.01). JY3 treatment significantly reduced TNF-*α* level compared to MCAO + CUMS rats (*P* < 0.05), whereas JY1 significantly reduced hippocampal TNF-*α* level (*P* < 0.05) and CIT10 significantly reduced hippocampal and prefrontal cortex TNF-*α* level (*P* < 0.05) (Figures 7(a)–7(c)). Serum and prefrontal cortex IL-1*β* levels were unchanged among groups (Figures 7(d) and 7(e)). Hippocampal IL-1*β* level was significantly increased in MCAO + CUMS rats compared to CON rats (*P* < 0.05), and JY3 significantly reduced IL-1*β* level (*P* < 0.05) (Figure 7(f)). The results suggested that the excess level of cytokines could be inhibited by administration of JY on PSD rat model.

### 3.7. JY Regulated the Expression of NF-*κ*B and I*κ*B-*α* in Hippocampal and Prefrontal Cortex

**Figure 8:**
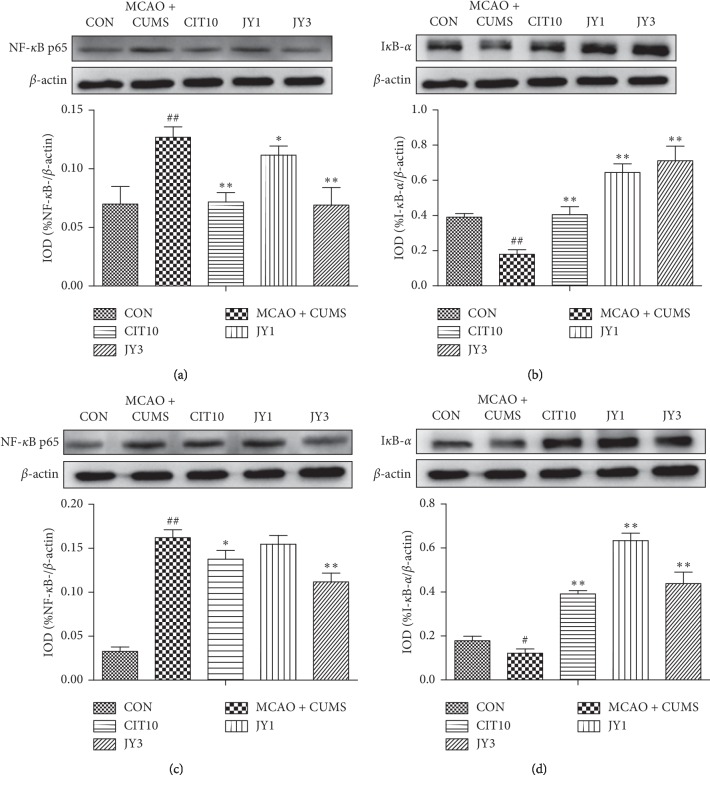
Effect of JY on the expression of NF-*κ*B and I*κ*B-*α* in prefrontal cortex and hippocampus of PSD rats. (a) NF-*κ*B expression in the prefrontal cortex; (b) I*κ*B-*α* expression in the prefrontal cortex. (c) NF-*κ*B expression in the hippocampus; (d) I*κ*B-*α* expression in the hippocampus. A representative blot and densitometric data for indicated bands are shown. Data are means ± SEM of triplicate experiments. ^#^*P* < 0.05,  ^##^*P* < 0.01 vs. CON. ^*∗*^*P* < 0.05,  ^*∗∗*^*P* < 0.01 vs. MCAO + CUMS.

The expression of NF-*κ*B was significantly increased in prefrontal cortex in MCAO + CUMS rats compared to CON rats (*P* < 0.01). CIT10, JY1, and JY3 treatment significantly decreased the expression of NF-*κ*B (*P* < 0.05, *P* < 0.01) (Figure 8(a)). The expression of I*κ*B-*α* was significantly decreased in prefrontal cortex in MCAO + CUMS rats compared to CON rats (*P* < 0.05). CIT10, JY1, and JY3 treatment significantly decreased the expression of I*κ*B-*α* (*P* < 0.01) (Figure 8(b)). The expression of hippocampal NF-*κ*B was significantly increased in MCAO + CUMS rats compared to CON rats (*P* < 0.01). CIT10 and JY3 treatment significantly decreased the expression of NF-*κ*B (*P* < 0.05, *P* < 0.01) (Figure 8(c)). The expression of hippocampal I*κ*B-*α* was significantly decreased in MCAO + CUMS rats compared to CON rats (*P* < 0.01). CIT10, JY1, and JY3 treatment significantly decreased the expression of I*κ*B-*α* (*P* < 0.01) (Figure 8(d)). The results suggested that JY could exert anti-inflammation effect by inhibiting NF-*κ*B/I*κ*B signaling on PSD rat model.

### 3.8. Quantifying the BrdU+/NeuN+ Cells in Dentate Gyrus of Hippocampus

**Figure 9:**
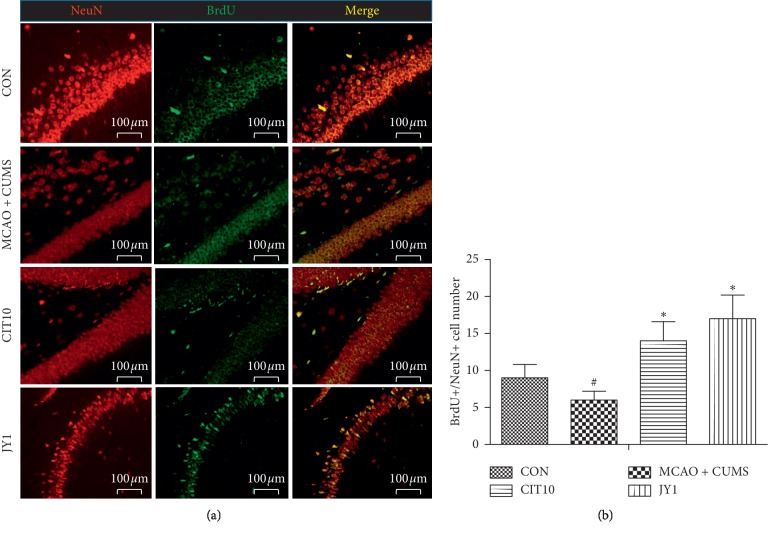
Representative confocal microscopy images of double-stained hippocampal section showing BrdU (green) and NeuN (red). (a) Representative confocal images for BrdU+/NeuN+ cells. (b) Quantification of BrdU+/NeuN+ cells. Data are means ± SEM. ^#^*P* < 0.05 vs. CON. ^*∗*^*P* < 0.05 vs. MCAO + CUMS.

To determine the effect of JY on the newly formed neurons in the dentate gyrus of hippocampus, the newly formed neurons were assessed by analysis of BrdU with the neuronal marker NeuN (Figure 9(a)). The number of BrdU+/NeuN+ cells was significantly decreased in the dentate gyrus of hippocampus in MCAO + CUMS rats compared to CON rats (*P* < 0.05). CIT10 and JY1 treatment significantly increased the number of BrdU+/NeuN+ cells in the dentate gyrus of hippocampus (*P* < 0.05) (Figure 9(b)). The results suggested that JY could significantly improve the neurogenesis of hippocampus on PSD rat model.

## 4. Discussion

PSD is the most frequent neuropsychiatric consequence, and experimental models may also pave the way for the discovery of novel therapeutic strategies [20]. The PSD model, which is developed by MCAO plus CUMS in our study, showed significant depressive behaviors, including decreased sucrose preference and motivation. Decreased sucrose preference in SPT is consistent with anhedonia, the major symptom of depression [21, 22], whereas the OFT measures the horizontal and vertical movement of rats for the evaluation of activity and curiosity [23]. The SPT and OFT are widely accepted behavioral paradigms for assessing pharmacological antidepressant activity [24]. Treatment with JY reversed the decreased sucrose preference and motivation in PSD rats the same as treatment with citalopram, indicating that JY could exert an antidepressant-like activity in PSD rats.

PSD is strongly associated with further worsening of physical and cognitive recovery, functional outcome, and quality of life [20]. The impairment of physical and cognitive function is thought to be the factor most closely associated with PSD development and severity [25]. It has been shown that the physical and cognitive impairments could be reversed by treatment with TCM via targeting multiple pathways [26]. In our study, we found that PSD rats exhibited both neurological impairments such as reduced asymmetry, strength, and decreased walking time, as well as impaired memory with MWMT test. BWT is a behavior test on assessing motor coordination after a stroke, and the CT and FGFT tests are behavior tests on assessing subjects for motor impairments [4, 27, 28]. The impairment of neurological and cognitive functions was observed in our PSD rat model, and treatment with JY significantly decreased the impairments, including improving grip force, decreasing asymmetry and beam crossing time. Lesions in the hippocampus are known to disrupt memory and spatial awareness [17]. It was shown that treatment with JY could improve the disrupted spatial memory in PSD rats.

Pathophysiology of PSD is complex and multifactorial, resulting from the combination of ischaemia-induced neurobiological dysfunctions and psychosocial distress [29]. An integrated view of the etiopathology of PSD posits the interlinking between monoamines, neuroinflammation, HPA axis, and neurogenesis as the common denominator [30, 31].

The central monoamine hypothesis proposes that imbalances in 5-HT, NE, and DA could result in depression [32]. Axons between the brainstem and cerebral cortex which contain these neurotransmitters can be disrupted during PSD, thus leading to imbalances in 5-HT, NE, and DA synthesis throughout the brain [33, 34]. In this study, neurotransmitters such as 5-HT, NE, and DA were assessed in prefrontal cortex, hippocampus, hypothalamus, and striatum of rats, which showed that the level of neurotransmitters was suppressed by CUMS exposure, which is consistent with previous studies. However, treatment with JY upregulated the levels of neurotransmitters. Certain 5-HT_1A_R ligands are currently used as a means of treating depression, such vilazodone and vortioxetine (SSRIs and partial 5-HT_1A_R agonists), or generalized anxiety disorder, such as buspirone (a partial 5-HT_1A_R agonist) [26]. Regulating ADRA2, associated with stress-dependent depression, may also improve treatment of a range of neuropsychiatric disorders [35]. We observed abnormal expression of 5-HT_1A_R and ADRA2 in the prefrontal cortex and hippocampal in PSD rats compared to CON rats, which could be also reversed by treatment with JY.

Depression is known to be linked with hyperfunctionality of the HPA axis [36], which primarily regulates physiological and psychological reactions to environmental changes [37]. Elevated stress hormone levels can be released as a result of chronic stress [38]. Impaired negative feedback in the HPA axis can cause levels of these stress hormones to continuously climb in a manner correlated with 5-HT system activity [39]. In this study, the level of serum CORT and ACTH was significantly increased in PSD rats. Hippocampal structure, acquisition of memory, and regulation of emotion could be affected by glucocorticoids [38]. We found that treatment with JY decreased the level of CORT and ACTH in the serum of PSD rats, consistent with reduced HPA axis hyperfunctionality. Glucocorticoids also have the potential to influence the structure of the hippocampus, as well as memory acquisition and emotional regulation [40]. Thus, JY could well regulate the functions of memory and emotion through HPA axis. GR is also most highly expressed in the hippocampus and can therein negatively regulate the HPA axis [41]. Altered HPA axis functionality is associated with both increased CORT level and decreased GR expression [42]. Chronic stress in animals is also known to reduce the plasticity and long-term potentiation of CA1 hippocampal neurons in a GR-dependent fashion, resulting in impairments to both learning and adaptation [43]. We found that CUMS treatment significantly decreased GR expression in the hippocampus of PSD rats, which could be reversed by JY treatment.

Increased levels of cytokines are also known to be linked to the pathophysiology of PSD [43]. Cerebral ischaemia leads to the production of increased proinflammatory IL-1*β* and TNF-*α*, which could further result in decreased 5-HT production, additionally promoting depression [44, 45]. The cytokines could modulate 5-HT metabolism and HPA axis functionality, further modulating the pathophysiology of depression [46]. Reducing CORT level and inflammation can enhance the synthesis of 5-HT [47]. We observed increased levels of TNF-*α* and IL-1*β* in the serum, prefrontal cortex, and hippocampus of PSD rats, in addition to elevated CORT levels. That is to say, JY treatment could significantly inhibit neuroinflammation in PSD rat model. Moreover, we detected the expression of NF-*κ*B and I*κ*B-*α*, which regulates inflammatory cytokine production, and found that JY was able to inhibit neuroinflammation in the prefrontal cortex and hippocampus in PSD rats through the inhibition of NF-*κ*B/I*κ*B signaling.

It has been shown that newly formed neurons in the dentate gyrus were detected by the increasing of BrdU+/NeuN+ cells [48]. Moreover, this study showed the decreased BrdU+/NeuN+ cells, while treatment with JY was associated with significant increase of BrdU+/NeuN+ cells in the dentate gyrus of hippocampus in PSD rats.

## 5. Conclusion

Our results indicate that JY could markedly exert antidepressant effects, also including improving neurological and cognitive functions in PSD rat model. These beneficial effects of JY may be involved in the modulation of levels of neurotransmitters and their receptors, the restoration of dysfunction in HPA axis, the inhibition of neuroinflammation, and the improvement of neurogenesis in hippocampus.

## Figures and Tables

**Table 1:** Information of Chinese herbs in JY.

Latin scientific name	English name	Pinyin name	Amount (g)
*Bupleurum abchasicum* Manden.	Radix Bupleuri	Chaihu	80
*Ziziphus jujuba* Mill.	Semen Ziziphi Spinosae	Suanzaoren	100
*Dens* Draconis	Dragon's Teeth	Longchi	200
*Polygala tenuifolia* Willd.	Radix Polygalae	Yuanzhi	80
*Lilium brownii* var. *viri* *du* *lum* Baker	Bulbus Lilii	Baihe	200
*Atractylodes macrocephala* Koidz.	Rhizoma Atractylodis Macrocephalae	Baizhu	60
*Triticum aestivum* L.	Fructus Tritici Levis	Fuxiaomai	200
*Angelica sinensis* (Oliv.) Diels	Radix Angelicae Sinensis	Danggui	60
*Acorus tatarinowii* Schott	Rhizoma Acori Tatarinowii	Shichangpu	80
*Pinellia ternata* (Thunb.) Makino	Rhizoma Pinelliae	Banxia	60
*Glycyrrhiza uralensis* Fisch.	Radix et Rhizoma Glycyrrhizae	Gancao	60
*Gardenia jasminoides* J. Ellis	Fructus Gardeniae	Zhizi	80
*Arisaema Cum* Bile	Arisaema cum Bile	Dannanxing	80
*Curcuma longa* L.	Radix Curcumae	Yujin	80
*Poria*	Poria Cocos	Fuling	100
*Fructus* Jujubae	Fructus Jujubae	Dazao	60

**Table 2:** Effect of JY on levels of NE, DA, and 5-HT in the prefrontal cortex, hippocampus, hypothalamus, and striatum of PSD rats; *μ*g/g tissue.

Brain area	Groups	NE	DA	5-HT
Prefrontal cortex	CON	42.96 ± 3.39	8.54 ± 1.57	0.58 ± 0.06
	MCAO + CUMS	31.84 ± 5.60	4.98 ± 0.51^##^	0.43 ± 0.03^##^
	CIT10	35.30 ± 2.35	6.15 ± 1.17^*∗*^	0.56 ± 0.06^*∗∗*^
	JY1	36.01 ± 3.64	5.94 ± 0.63^*∗*^	0.56 ± 0.05^*∗∗*^

Hippocampus	CON	31.90 ± 4.74	8.61 ± 0.65	0.70 ± 0.09
	MCAO + CUMS	23.05 ± 4.09^#^	6.53 ± 0.30^##^	0.51 ± 0.11^#^
	CIT10	25.71 ± 8.01	7.50 ± 1.03	0.67 ± 0.05^*∗*^
	JY1	30.63 ± 5.03^*∗*^	7.44 ± 0.50^*∗∗*^	0.68 ± 0.06^*∗∗*^

Hypothalamus	CON	42.54 ± 2.44	7.16 ± 0.46	0.50 ± 0.07
	MCAO + CUMS	31.63 ± 4.83^##^	5.54 ± 0.62^##^	0.38 ± 0.04^##^
	CIT10	39.79 ± 3.58	6.73 ± 0.77^*∗*^	0.45 ± 0.03^*∗∗*^
	JY1	40.07 ± 1.62^*∗∗*^	6.72 ± 1.22^*∗*^	0.44 ± 0.03^*∗*^

Striatum	CON	51.88 ± 8.41	9.96 ± 1.58	0.70 ± 0.12
	MCAO + CUMS	40.79 ± 8.17^#^	8.00 ± 0.84^#^	0.58 ± 0.08^#^
	CIT10	50.86 ± 7.25^*∗∗*^	9.66 ± 2.17	0.60 ± 0.16
	JY1	51.51 ± 4.97^*∗*^	8.33 ± 1.17	0.64 ± 0.14

Data are expressed as mean ± SEM. *n* = 7–10. ^#^*P* < 0.05,  ^##^*P* < 0.01 compared to CON group. ^*∗*^*P* < 0.05,  ^*∗∗*^*P* < 0.01 compared to MCAO + CUMS group.

## Data Availability

The data used to support the findings of this study are available from the corresponding author upon request.
